# From IR Images to Point Clouds to Pose: Point Cloud-Based AR Glasses Pose Estimation

**DOI:** 10.3390/jimaging7050080

**Published:** 2021-04-27

**Authors:** Ahmet Firintepe, Carolin Vey, Stylianos Asteriadis, Alain Pagani, Didier Stricker

**Affiliations:** 1 BMW Group Research, New Technologies, Innovations, 85748 Munich, Germany; c.vey@alumni.maastrichtuniversity.nl; 2 Department of Informatics, University of Kaiserslautern, 67653 Kaiserslautern, Germany; Didier.Stricker@dfki.de; 3 Department of Data Science and Knowledge Engineering, Maastricht University, 6211 TE Maastricht, The Netherlands; Stelios.Asteriadis@maastrichtuniversity.nl; 4 German Research Center for Artificial Intelligence (DFKI), 67653 Kaiserslautern, Germany; Alain.Pagani@dfki.de

**Keywords:** computer vision, augmented reality, object pose estimation, point clouds, deep learning

## Abstract

In this paper, we propose two novel AR glasses pose estimation algorithms from single infrared images by using 3D point clouds as an intermediate representation. Our first approach “PointsToRotation” is based on a Deep Neural Network alone, whereas our second approach “PointsToPose” is a hybrid model combining Deep Learning and a voting-based mechanism. Our methods utilize a point cloud estimator, which we trained on multi-view infrared images in a semi-supervised manner, generating point clouds based on one image only. We generate a point cloud dataset with our point cloud estimator using the HMDPose dataset, consisting of multi-view infrared images of various AR glasses with the corresponding 6-DoF poses. In comparison to another point cloud-based 6-DoF pose estimation named CloudPose, we achieve an error reduction of around 50%. Compared to a state-of-the-art image-based method, we reduce the pose estimation error by around 96%.

## 1. Introduction

In the last decades, object pose estimation has been an ongoing research field due to its significance in robotics, autonomous driving, and Augmented Reality (AR). In robotics, the correct estimation position and orientation for assembly, disassembly, pick, and place are crucial. This is typically done by RGB-D sensors built inside the robot. In autonomous driving, the same information is required to effectively handle other road users like cars, bicycles, or pedestrians. For this purpose, expensive equipment like LiDAR sensors is deployed for a 3D perception and consequent pose estimation of various objects. AR requires the 6-DoF pose of objects for applications like remote interaction. AR-enabled devices use RGB-D sensors built into smartphones or AR glasses. Another important aspect of object pose estimation in AR is the computation of the 6-DoF pose of the AR glasses themselves to ensure a correct augmentation with the virtual objects. When dealing with the problem of tracking AR glasses inside a moving car, an interesting and efficient alternative is infrared (IR) cameras due to their light invariance property. In addition, inside-out tracking is difficult as sensors in an AR glasses capture mostly dynamic features due to the car movement. Thus, in the car context, cameras inside of AR glasses register a limited amount of static features to conduct tracking. In this case, cameras built inside the car are preferable to perform outside-in tracking of the AR glasses.

Expensive depth sensors are usually deployed to enhance robustness for pose estimation. Cheap sensors like RGB or IR sensors would be preferable to use if pose estimation of sufficient accuracy would be achievable. Multiple solutions for object pose estimation have been proposed in the Computer Vision literature, making use of RGB information [1,2,3,4]. However, the vast majority of these solutions are highly sensitive to varying lighting conditions and shadows. IR images are a potential alternative, but they are more challenging due to the lack of color information. Nonetheless, IR cameras have the core advantage of providing images with lighting conditions less dependent to external parameters. This is especially desirable in case of AR glasses deployment inside cars to enable use cases like AR navigation. In this work, we evaluate if point clouds derived through IR images in a semi-supervised manner with subsequent AR glasses pose estimation can improve pose estimation accuracy compared to pose estimation directly done on IR images. Our evaluation is based on a multi-view AR glasses IR dataset called HMDPose [5].

**Figure 1:**
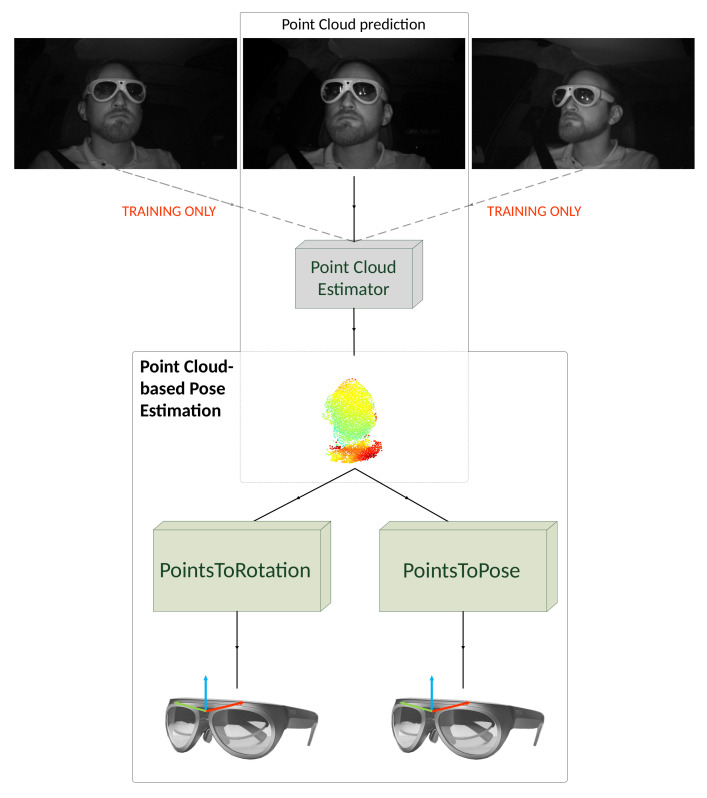
Our full object pose estimation pipeline. We train our point cloud estimator on three images and need one image during testing only to predict a point cloud. We use the point cloud for both our pose estimation methods.

This paper proposes two approaches to estimate object pose with point clouds, generated through single IR images only. We develop and utilize a monocular, semi-supervised point cloud generator to generate point clouds on single IR images to facilitate this. The point cloud estimator is trained on three multi-view infrared images of all four AR glasses simultaneously of the HMDPose dataset and does not require depth ground truth during training (Figure 1).

In more detail, our contributions are:

- We present a novel monocular semi-supervised point cloud generator trained on multiple images to generate point clouds on single IR images.
- We introduce a Deep Learning-based method to estimate the object orientation from a 3D point cloud (“PointsToRotation”), directly and efficiently regressing the orientation.
- We propose a larger hybrid model combining Deep Learning for seed point generation and a voting-mechanism to estimate the full 6-DoF pose from point clouds (“PointsToPose”).
- We evaluate our approaches on the HMDPose dataset [5] and compare our results with the state-of-the-art image-based approach “RandomizedBins” [6] and the point cloud-based “CloudPose” [7] method, showing a reduction of the pose error by around 96% and 50%, respectively.

In the remainder of the paper, we discuss the related work on object pose estimation in Section 2. We present our point cloud estimator and two pose estimation techniques in Section 3. In Section 4, we discuss our evaluation on the HMDPose dataset.

## 2. Related Work

Object pose estimation in Computer Vision is traditionally done either based on RGB images or with depth information, usually coupled with RGB data in the form of RGB-D input. Given that depth information usually necessitates the use of dedicated hardware, image-only approaches for pose estimation have received significant attention over the last years [1,3,4,6,8].

### 2.1. Image-Based Pose Estimation

Many image-based object pose estimation methods have been developed in recent years. Traditional methods mainly focus on extracting hand-crafted features from the input image, with subsequent feature matching of a 3D model and finally using the 2D-3D point correspondences to perform Perspective-n-Point (PnP) [9,10]. Because handcrafting features is time-consuming and prone to errors, Deep Learning-based approaches have gained popularity and outperform traditional approaches [1,3,4,8]. Image-based Deep Learning methods can be categorized into feature-based and appearance-based approaches. Appearance-based methods use the complete pixel information present in an image. In contrast, feature-based methods require the definition of features on the target object to be later tracked. Recent feature-based Deep Learning methods use Deep Neural Networks to estimate the objects’ keypoints and combine them with PnP, partly relying on traditional methods [1,2,11]. Peng et al. [1] provide a state-of-the-art approach based on keypoint regression and further PnP execution. A novelty in their approach is the regression of 2D vectors from each pixel to the object’s predefined keypoint positions. Based on this vectorfield output, they conduct a voting-based method to estimate to most probable keypoint position and its uncertainty as a covariance matrix. Then, they perform PnP.

Appearance-based methods directly regress the pose based on the input image [3,4,6,8]. Berg et al. propose a regression via classification pipeline for pose estimation, directly building upon input RGB images [6]. Their regression via classification approach introduces the usage of several discrete data representations simultaneously to improve neural network learning in comparison to classifications utilizing a single representation. We utilize this method for our evaluation. It is the most recent, appearance-based method, which we can benchmark on the HMDPose dataset without requiring further ground truth information like keypoints.

### 2.2. Depth-Based Pose Estimation

#### 2.2.1. RGB-D-Based Methods

Depth-based pose estimation approaches like RGB-D-based methods usually result in better estimation accuracy than image-based methods [12,13,14] but are often tied to more costly hardware. Traditional approaches use predefined 3D features extracted from RGB-D input to match estimated features [15,16]. In addition to methods that estimate the pose directly from RGB-D data [3], some recent approaches fuse information from RGB or depth maps before estimating a pose [17,18]. Wang et al. [18] fuse color embeddings of the RGB input and geometry embeddings of the depth map to a dense feature representation. The dense representation is subsequently used to predict poses per pixel, including a prediction confidence. Finally, a pose per image is generated thorugh voting. Xu et al. [12] follow a similar approach with the extension of incorporating pixel-pair pose predictions and object model-based dense corresponding mapping.

#### 2.2.2. Point Cloud-Based Methods

Applying Deep Learning networks based on point cloud input for various Computer Vision tasks is continuously gaining popularity due to higher performance and more efficient depth information representation [19,20,21].

For pose estimation, some approaches exist using RGB-D inputs to generate point clouds in an intermediate step for further object pose estimation [7,12,18,22], head pose estimation [23,24], hand pose estimation [25,26,27], and camera pose estimation [28,29]. Gao et al. [22] propose a point cloud-based object orientation regression approach. In a more recent work, they adapt this approach to full 6-DoF object pose estimation [7]. They feed the point clouds generated through RGB-D inputs into two CNNs, where each subnetwork predicts either the position or the orientation. We benchmark this method by training the part of the proposed network, which predicts the pose based on point clouds.

However, point clouds have been mostly used as an intermediate depth representation step only. Qi et al. [30] propose a Deep Neural Network for 3D object detection and scene parsing, working directly on point clouds. The network can predict the semantic class and a bounding box with the center, scale, and 1D-heading angle per object in the scene. Unlike our approach, they do not perform object pose estimation. The network’s backbone is based on set abstraction and feature upsampling layers as proposed in PointNet++ [20]. The Hough voting module is a vital component of the method. While the object centroid can be hard to regress due to its considerable distance to the point cloud’s surface points, the voting mechanism significantly simplifies this task.

Coming from real-world restrictions, situations like the deployment of AR glasses in cars exist, where light invariance and cost efficiency are crucial. Despite IR images containing less information than RGB images due to their grayscale nature, they provide the core advantage of being light invariant. To the best of our knowledge, Computer Vision research has not investigated methods for pose estimation using point clouds directly generated from IR images yet. Consequently, our pipeline decreases the effect of lighting variance in a car by estimating point clouds from IR images for AR glasses pose regression.

## 3. Methodology

Ongoing developments in Deep Learning are solving an increasing number of challenging Computer Vision problems, including object recognition and pose estimation. Neural network architectures are deployed for 3D object detection and classification as well as scene semantic parsing. For the object pose problem, some approaches estimate the bounding box center for translation and heading for rotation [30,31], the rotation being defined with only a one-dimensional heading angle. However, in our case, we aim for three-dimensional rotation information. The specialized PointNet++ layers [30] have been proven to extract valuable information from point clouds in the form of so-called critical points or seed points. From an object classification point of view, these points represent the minimum set of points, still providing enough information to identify an object [19]. Thus, this set is a good descriptor for a point cloud. We provide two Deep Neural Network architectures called PointsToRotation (P2R) and PointsToPose (P2P), whose backbones are based on these layers. With our P2R architecture, we can directly regress 3D rotation from the target object’s found seed points. Our P2P method extends the first method by a voting module and a translation estimation. Our two methods require point clouds, which we first estimate with our custom, multi-view trained, monocular point cloud estimator.

### 3.1. Point Clouds Generation

To generate point clouds, as well as for training pose estimators, we make use of a multi-view IR-based dataset, namely HMDPose [5]. No other significant datasets are widely available to the research community containing multi-view IR images of objects. HMDPose is a dataset consisting of various IR images of four different AR glasses worn by 14 subjects, appended with 6-DoF ground truth pose annotations. The dataset contains synchronized image triples per pose. The images come from cameras positioned to the left, right, and directly in front of the driver wearing the AR glasses.

**Figure 2:**
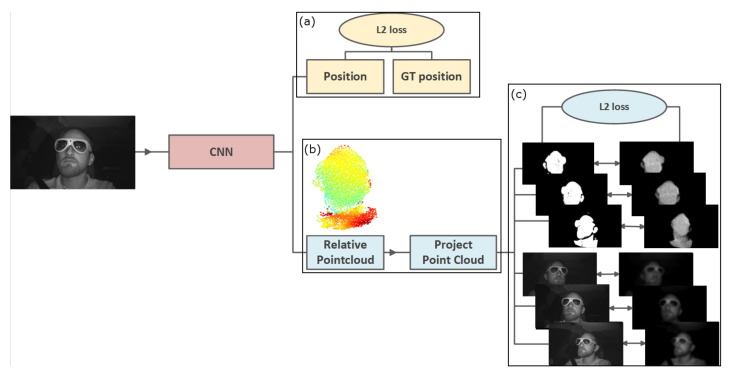
An overview of our overall architecture. (**a**) We estimate the translation based on the ground truth. (**b**) The point cloud estimation is performed with self-supervision based on (**c**) pre-generated masks of the target objects and the intensities of the original images.

Our point cloud estimator is a semi-supervised Deep Learning approach. The network learns by projecting the generated point cloud onto other frames of the same scene captured from a different perspective than the input frame. The projection per view is compared to a ground truth foreground mask of this perspective’s respective image. By minimizing the offset between projection and ground truth per view, the network can produce a 3D representation that is suitable for all views. The network uses ground truth translation and generated masks for self-and semi-supervision. Figure 2 illustrates a full overview of the proposed pipeline, including the losses.

#### 3.1.1. Mask Generation

**Figure 3:**
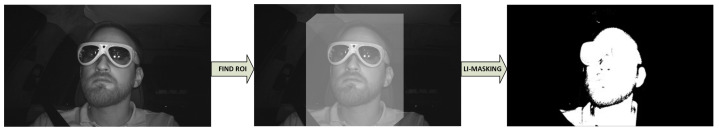
Mask generation pipeline. From left to right: determine region of interest based on projection of rendered cuboid and Li-Thresholding.

In the first pre-processing step, we generate masks to supervise our network. The supervision is based on the comparison of the generated masks with the 2D projections of predicted 3D points. The masks assure that the network learns to omit unnecessary background information and focuses on the foreground information from the input images for point cloud generation (Figure 3).

First, we define the region of interest in the image by rendering a cuboid based on the current ground truth pose given by the HMDPose dataset [5]. The cuboid approximates the upper body and filters noise visible from the background. The cuboid has the sizes width = 17.8 cm, height = 26.4 cm and depth = 25.7 cm. In the next step, we apply Li-thresholding [32,33], a method to separate the foreground from the background by iterative cross-entropy minimization. This method is effective due to the foreground being much lighter than the background, and thus the object can be separated easily.

#### 3.1.2. Neural Network for Point Cloud Estimation

**Figure 4:**
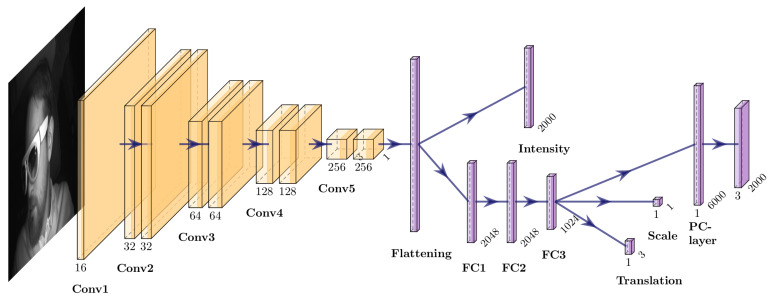
An overview of the CNN architecture. We use convolutional layers to extract features from the input image. The number of filters is doubled per convolutional block. The kernel size changes between one and three, starting with three in the first layer of the first convolutional block. While color information is directly regressed from the flattened feature vector, a decoder processes the feature vector before scale, translation and point cloud are predicted.

We propose a Deep Neural Network to estimate the object’s intensity, shape, and location in a given input image. We train a CNN to predict point cloud information in a semi-supervised manner, based on positional ground truth information and multi-view camera images. The predicted point cloud involves positional and intensity information per point. The training on intensity and scale are crucial to stabilize and improve the point cloud quality during training. The network benefits from the intensity and scale information to estimate 3D points on the correct 3D position during training. To assess the accuracy of the predicted point cloud, we transform and project it into the other camera views using an adapted version of the differentiable point cloud renderer proposed by Insafutdinov et al. [34]. Figure 4 shows a detailed overview of the utilized CNN.

One input image of a given triple is fed through the network. First, the encoder extracts features from the input images. Extracted features are flattened and further used to regress the intensity information per point directly. Having these encodings, we use three further fully connected layers to predict the overall translation of the object $t^{\prime }$, a relative point cloud of 2000 points with *x*, *y*, *z* coordinates, and scaling factor *s* to control the points’ size. Note that the intensity information is already present in the input image. This is why we directly derive this information from the convolved features.

The center of the point cloud and its prediction is positioned in the relative center per point cloud, since the predicted 3D points determine the offset to the predicted translation value. This approach allows us to separate shape and positional information and improve them in separate loss terms. The CNN predicts a point cloud to be projected into the different camera views to be used for our mask projection loss $L_{mp}$. For this projection the camera intrinsics *C* and camera extrinsics *T* are required. This info is taken from the HMDPose dataset. We homogenize predicted points $p^{s}$ and receive the points $p_{h}^{s}$. We then add the ground truth translation *t* per point. For a given camera among the left, right, and center cameras, we compute camera coordinates $p^{c}$ using the inverse camera intrinsics $C^{-1}$:

(1) $$p^{c}=C^{-1}*\left(p_{h}^{s}+t\right)$$

Then, we can compute world coordinates $p^{w}$ by multiplying them with the inverse camera extrinsics *T*:

(2) $$p^{w}=T^{-1}*p^{c}$$

Taking into consideration the ground truth translation and the predictecd depth information per point, we compute image coordinates for the other camera views by projecting $p^{w}$ to the image plane per view. The remaining projection pipeline follows the differentiable point cloud projector proposed in [34].

We make use of three different loss terms to improve the predictions of the network. The loss terms for the mask projection and intensity mask projection are adapted from Insafutdinov et al. [34], with the addition of normalization to each term.

**Figure 5:**
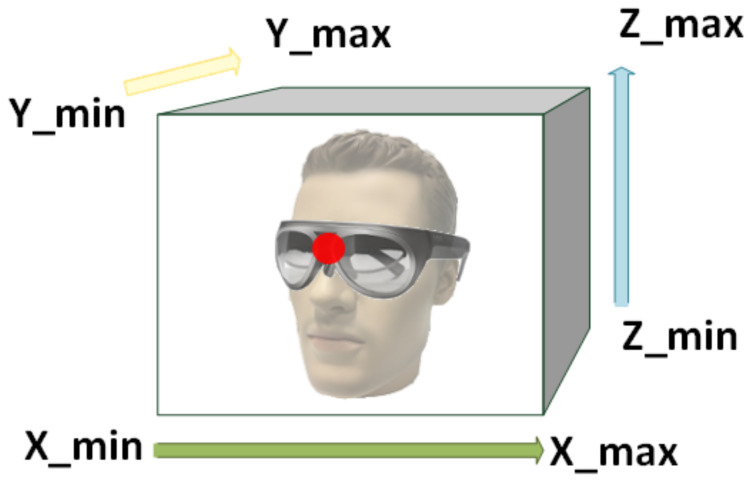
Prediction relative to object box. The predicted translation (red) is relative to a predefined object box. The borders of this box have been determined as the minimum and maximum translation values per axis based on the ground truth annotations.

- **Translation loss**$L_{t}$ The accuracy of the positional prediction is measured as the $L_{2}$ loss between ground truth translation and the predicted translation after conversion to the object box. This box’s borders are set as the minimum and maximum translation values per axis based on the ground truth annotations. This is visualized in Figure 5.
- **Mask projection loss**$L_{mp}$ The mask projection loss measures the $L_{2}$ loss between the predicted point cloud’s projection and the generated masks. The ground truth mask is smoothed with a Gaussian kernel with filter size K=3 and variance Σ=s, where *s* is linearly increased from 0.2 to 1.0 during training. The image resolution normalizes the loss.
- **Intensity mask projection loss**$L_{cmp}$ This loss term measures the discrepancy between the intensity mask projection and the intensity ground truth information by applying an $L_{2}$ loss. Therefore we supplement the predicted intensity information to the point cloud prediction before projection. Intensity masks are generated by masking the original input images with the generated masks according to Section 3.1.1. The following procedure is similar to the mask projection loss.

The overall loss is computed as a weighted average of the three loss terms:

(3) $$L=a*L_{t}+b*L_{mp}+c*L_{cmp}$$

**Figure 6:**
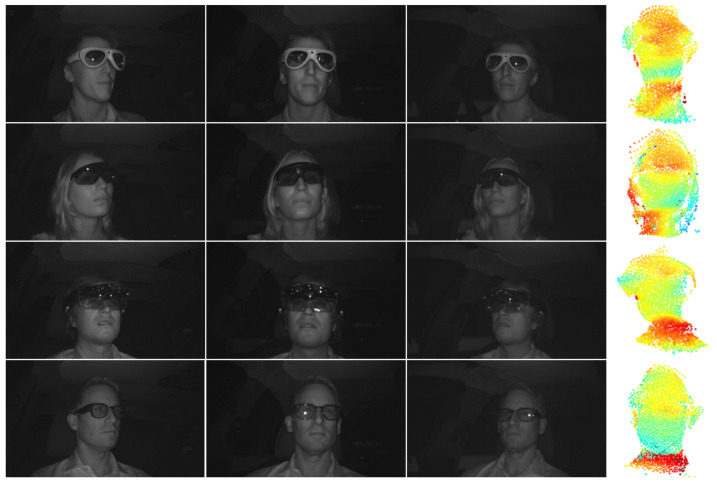
Four example triples with the estimated point cloud, predicted by the central image only. Each row visualizes an example triple for a different person, wearing different glasses and the corresponding point cloud. The lighter the blue color of 3D point, the more the relative distance of a particular 3D point.

*a*, *b* and *c* is for the weighting of the loss terms. We set *a* = 0.2, *b* = 0.2 and *c* = 0.6, which we derive from experiments. This weighting assures that the crucial translation loss $L_{t}$ and the mask projection loss $L_{cmp}$ have more weight in the overall loss. The intensity mask projection loss $L_{mp}$ assists them in strengthening the point cloud estimation quality. Figure 6 shows example results of the point cloud estimation. Each row shows a point cloud estimation of a different person wearing a different type of glasses. The point clouds are shown from slightly different perspectives. The point clouds are visualized in the typical color-coding scheme for depth information. Close points to the camera are blue, whereas more distant 3D points are red.

#### 3.1.3. Training Details

We use HMDPose for training and testing [5]. Our training, validation, and test split is 94% for training and 3% for validation and test set each. Before splitting, the dataset is being shuffled. All images are rescaled to a quarter of their original input size of 1280×752 pixel to 320×188 pixel and normalized. Decreasing the original input resolution is required due to computational limits. The network is trained using Adam Optimizer with a learning rate of 0.0001 and standard momentum parameters. We train the model by randomly selecting one of the three views as input. The predicted point cloud is projected to a voxel grid of 160×94×160 for width, height, and depth, respectively.

#### 3.1.4. Difference in Training and Testing

While the training procedure relies on multi-view input images and ground truth positional information, we only need one input image of an arbitrary view at test time. During training, the network learns to produce point cloud information based on one input image, the other two views have only been used for semi-supervision. Since the predicted translation is relative to the point cloud center, we compute absolute translation values according to the predefined object box during test time.

### 3.2. Pose Representation

Based on the point clouds generated with our point cloud estimation approach, we perform 6-DoF pose estimation. We represent translation in Euclidean space and rotation via unit quaternions for our Deep Learning-based pose estimation approaches. We use quaternions as our format for representing rotations due to their successful usage compared to Euler angles and rotation matrices in previous work [35,36,37].

### 3.3. PointsToRotation Network

We introduce a Deep Neural Network called PointsToRotation (P2R) based on a PointNet++ [30] backbone and a rotation estimation module. The input point cloud is processed by four set abstraction and two feature propagation layers. These layers were introduced by Qi et al. [30], specially designed to handle point set input. Stacking these layers by iteratively abstracting the input point set and then propagating the learned features, allows finding so-called seed points or critical set. A set abstraction layer is able to group an input point set based on Furthest Point Sampling and ball region clustering. Applying a PointNet afterwards helps to extract feature information. Each group found by the set abstraction layer corresponds to a local region of the input point cloud, including centroid and surrounding local feature information. Repetitive use of this type of layer helps to abstract the input point cloud and to decrease the number of points while extracting deep features for selected points. A feature propagation layer addresses the problem of obtaining deep point features for all points, based on the found features during the set abstraction step. The propagation is realized through skip connections between the original input set and the abstracted set as well as K-nearest neighbor interpolation.

**Figure 7:**
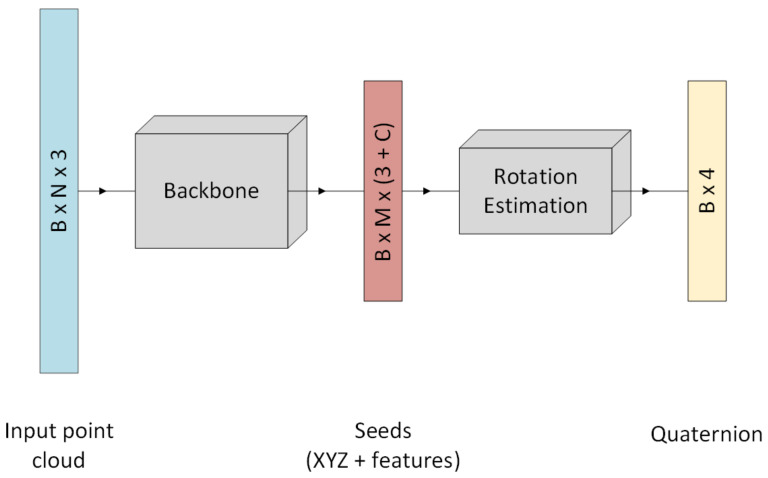
Overview of P2R-Net architecture. N is the number of points, B refers to the batch size of the network, M is the number of seed points and C is the number of additional features found by the network.

Based on the backbone features, we regress 3D rotation information from found seed points using several 1×1 convolutional layers and dense layers. Figure 7 visualizes the complete network architecture.

The backbone is built of four set abstraction layers and two feature propagation layers. The progressive involvement of increasingly large regions of the point set allows for hierarchical point set feature learning [20]. Table 1 shows the exact parameters for the backbone layers.

The ball radii for the set abstraction layers have been selected concerning the average head size of drivers and are given in meters. The ball radius parameter grows with the depth of the network from 0.02 to 1.0. The final set abstraction layer incorporates all points, such that the overall rotation of the object depends on the features of all found seed points.

In Table 2, the individual input and output tensor sizes per backbone layer are listed.

The backbone used for P2R subsamples 512 seed points with a 256-dimensional feature vector from 2000 input points with original feature dimension of 3. Thus, the proposed approach keeps 25.6% of the input points. The features learned by the backbone are then fed into a rotation estimation module, which consists of three 1×1 convolutional layers with batch normalization and ReLU activation function. While the first two layers use 256 filters each, the last layer works with 4 filters. The output is flattened and processed by two dense layers with 512 and 4 output nodes, respectively. The result is a four-dimensional vector representing a quaternion. Our rotation loss $L_{rot}$ normalizes the predicted quaternion and compares it to the ground truth quaternion using $L_{2}$ loss. Finally, the loss is normalized by the batch size.

### 3.4. PointsToPose Network

Building upon P2R, we propose another Deep Neural Network’s performance, which expands the proposed network with a voting module and a translation estimation. We call this approach PointsToPose (P2P). The pipeline is inspired by VoteNet [30], where the authors arguein favor of using 3D Hough voting to predict the centroid of 3D bounding boxes, as this point is not part of the surface, potentially acquired by a depth sensor. This results in more accurate bounding box predictions for the scene’s objects than direct regression of the bounding box positions.

**Figure 8:**
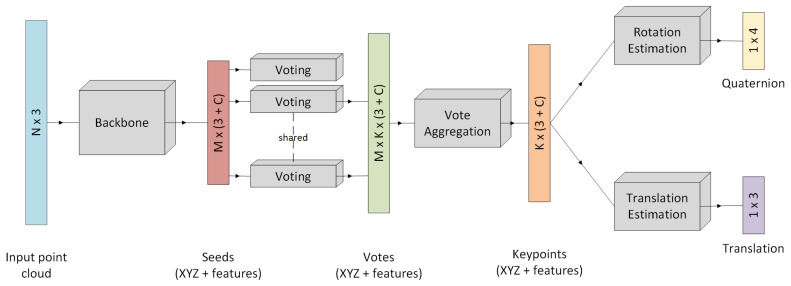
Overview of P2P-Net architecture. N is the number of points, M refers to the number of seeds found by the backbone and K is the number of predefined keypoints.

Compared to VoteNet [30], our input point cloud involves only ten percent of the number of points. VoteNet extracts 1024 seed points from the input point cloud with 20,000 points. A 256-dimensional feature vector characterizes each seed. The input point cloud amount is identical to the description in Section 3.3, starting with 2000 input points and generating 512 seed points. This approach aims to find seed points in the input point cloud and let the seeds vote for predefined keypoints *K*. Keypoint and voting-based object detection algorithms have proven to be successful when working with 3D point clouds. Especially the definition of keypoints benefits pose estimation when dealing with occlusions and truncation [1]. The pose estimation is assumed to be more efficient when predicted from keypoints instead of seeds. While the seed selection is heavily dependent on the input data, the definition of keypoints is independent of the input. However, it results in the same point distribution for every possible pose of the object. The keypoints are defined by subsampling a combined 3D model of a human head with glasses with Furthest Point Sampling. For this purpose, we fuse a 3D model of a synthetic male human head with a 3D model of glasses. These keypoints are the ground truth annotations for the votes. The network pipeline for this approach is shown in Figure 8.

At first, we select seed points from the input point cloud. In the next step, each seed point votes for each of the predefined keypoints. After aggregating the votes, rotation and translation information are regressed.

The loss of this network architecture is based on three different loss terms: rotation loss $L_{rot}$, translation loss $L_{trans}$ and vote loss $L_{vote}$. The rotation loss is computed in the same manner as for P2R. The translation loss refers to the sum of the predicted relative translation and the translation of the input point cloud. We use $L_{2}$ loss to compute the difference from ground truth translation. The vote loss supervises keypoint predictions (votes) with the ground truth position of this keypoint based on the $L_{2}$ loss. The final loss function is comprised according to Equation (4):

(4) $$L=a*L_{rot}+b*L_{trans}+c*L_{vote}$$

*a*, *b* and *c* is for the weighting of the loss terms, where we set *a* = 0.2, *b* = 0.2 and *c* = 0.6. The higher weighting of the vote loss ensures a valid base for the rotation and translation estimation. The weightings are derived through experiments.

## 4. Evaluation

### 4.1. Dataset and Evaluation Metrics

We conduct the training and evaluation of our approaches on the HMDPose dataset [5]. HMDPose is a large-scale IR dataset with AR glasses pose annotations, containing of around 3 million images, resulting in 1 million image triples. The datasets consists of four different AR glasses models, worn by 14 different subjects. It includes the four glasses models Everysight Raptor, Microsoft Hololens 1, North Focal Generation 1 and Mini Augmented Vision. In the evaluation of our paper, we refer to the Everysight Raptor as EVS, Hololens 1 as HOLO, North Focal Generation 1 as NORTH, the Mini Augmented Vision glasses as MAV and all glasses combined as ALL for readability. There are around 250,000 image triples per glasses model available.

To compare our two methods P2R and P2P with the two selected state-of-the-art approaches, we define the following metrics for further benchmarking of our results: Mean Absolute Error (MAE), Root Mean Squared Error (RMSE) and Balanced Mean Angular Error (BMAE). The BMAE considers the unbalanced amount of different head orientations by introducing section definition [38,39,40]:

(5) $$\mathrm{BMAE}:=\frac{d}{k}\sum_{i=1}\phi_{i,i+d},i\in dN\cap \left[0,k\right],$$

The metric divides the range of movement *k* into sections *i* with sizes *d*. This leads to extreme and rare poses being weighted equally to frequent and regular poses. $\phi_{i,i+d}$ is defined as the average angular error. We define the section size *d* as 5 degrees and the range size *k* as 180 degrees. For the position estimation of our second approach introduced in Section 3.4, we use the $L_{2}$ loss for the position error on all axes separately and together.

### 4.2. State-of-the-Art Object Pose Estimation Methods

#### 4.2.1. Image-Based Method

**Figure 9:**
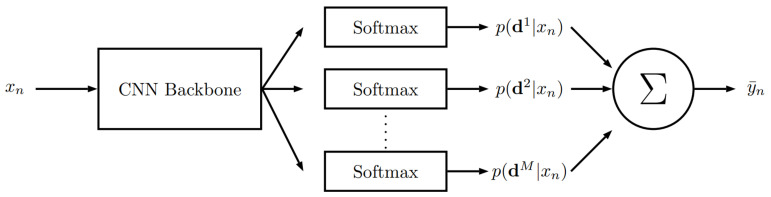
Overview of the RvC-based pose estimation method [6]. $p(d^{M}|x_{n})$ depicts the softmax layer outputs per class $D^{m}$ for an input $x_{n}$. The outputs are being combined using an ensemble average to obtain a final pose estimation.

In order to compare our methods to state-of-the-art algorithms, we first selected a recent image-based direct pose estimation method based on classification [6]. Berg et al. implement and evaluate various techniques for discrete class representation creation for regression via classification (RvC). They propose the usage of several discrete data representations simultaneously to improve neural network learning. Regarding head pose estimation, the most promising discrete class representation method is “RandomizedBins”. In this case, they introduce a set amount of class intervals $D^{m}$ in contrast to traditional RvC approaches with one class interval. Within each interval, they randomly sample *L* bins of varying width to maximize diversity between different discretizations $D^{m}$. Therefore, target values that do not belong to any of the chosen classes are assigned to the nearest neighbor in the sample. They utilize ResNet50 [41] as a backbone with additional fully connected layers for each estimated value. They use *M* fully connected layer for each class $D^{m}$ and *L* fully connected layer per class with a softmax layer for all outputs. Figure 9 shows the overall architecture.

The original implementation is focused on orientation estimation only, which we adjust to 6-DoF pose estimation. We do this by introducing discretizations for the position equivalent to the orientation. Therefore, we estimate values for classes of six different outputs instead of the initial three outputs for orientation. We train this method on the HMDPose dataset by setting the parameters for the method identical to the original implementation. Thus, we set L=40 random bins per class *M*, where we set M=30, and randomly sample them by dividing the label ground truth range in steps of 0.01 degree and 0.01 cm. For training the network, we use the full images with resolution 320×188 as input. We use the Adam optimizer with an initial learning rate of α=0.0001. Our training, validation and test split is 94/3/3, which is identical to the training of the other approaches, including the point cloud estimator. In the remainder of the paper, we refer to this approach as “RandomizedBins”.

#### 4.2.2. Point-Cloud-Based method

We additionally select a point cloud-based object pose esitmation approach by Gao et al. [7] named CloudPose for benchmarking. In the original work, the authors build upon semantic segmentation and RGB-D data to derive point clouds as an intermediate representation. Then, they peform 6-DoF pose estimation based on the point clouds. A network named “BaseNet” is being used for translation and orientation estimation seperately. The network is based on PointNet [19]. For rotation estimation, they utilize the axis-angle representation and deploy the geodesic loss function. During evaluation on the defined metrics, we convert the axis-angle predictions to Euler angles.

We benchmark this method based on on our generated point clouds and the ground truth pose labels. We use the identical training, validation and test split that we used to train our P2R and P2P methods. The parameters are kept as in the original implementation for comparabilty.

### 4.3. Orientation Results

After training the RandomizedBins [6], CloudPose [7], P2R, and P2P methods for 30 epochs each, we evaluate the translation and orientation error on the defined metrics.

Table 3 shows the error of the four methods on the defined metrics for the orientation on all axes individually and on average.

#### 4.3.1. Results on Objects per Method

The benchmarked RvC method RandomizedBins shows similar errors on the individual glasses, while ALL shows errors mostly on the higher end compared to the individual glasses. RandomizedBins performs best on MAE for EVS and NORTH, with an average MAE of 9.90° and 9.41°, both being the smaller glasses types in the dataset. All other objects individually and combined result in similar MAE averages. On the yaw, a considerably high error among all metrics and and all objects is observable, which is the heading angle of the glasses. The BMAE is significantly higher for ALL compared to individual glasses regarding the yaw with 48.49° and average with 28.15°, pointing towards a less effective pose estimation accuracy in extreme poses when all glasses combined. In general, this method performs better when trained on individual glasses.

The errors for CloudPose are close among the individually trained and combined glasses models. The MAE is below 1.12° on average, which is the highest for the smallest glasses model NORTH. The RMSE on average is between 1.24° and 1.45°, with the lowest value for ALL and HOLO, and the highest for NORTH. In return, the BMAE is lower for NORTH with 1.98° in contrast to 3.04° for EVS. This show that NORTH performs better in predicting extreme poses.

The errors for P2R are comparable in case of individually trained and combined glasses. The MAE error of the P2R algorithm is lower than 0.59° on all axes for all glasses models. For HOLO, we achieve the lowest errors with an average error of 0.42° overall on the MAE. NORTH results in the highest error for MAE. The BMAE shows the good estimation quality even for difficult cases like extreme poses among all objects with the exception of NORTH, being the smallest model in the dataset. NORTH results in more than twice as high error on the BMAE with an average of 2.47° in contrast to all other glasses. Larger glasses models like HOLO and MAV perform better with an average BMAE of 0.71° than the smaller ones. The results for ALL are as expected, as they are in the mid-range considering all other glasses individually.

Our P2P method performs similarly to the P2R method. There is little difference observable among all objects. P2P results in average estimation accuracy on ALL compared to the objects individually. On the RMSE metric HOLO and EVS result in the lowest overall error with average errors of 0.65°. The smallest glasses model NORTH results in the highest error compared to the other object individually on all metrics. The error on ALL is in the mid-range compared to the individual objects again with an average RMSE of 0.76°. On the BMAE metric, P2P performs best on the largest model HOLO with an average error of 0.72°, while achieving an average error of 1.68° for the smallest model NORTH. In general, P2P improves with larger AR glasses size.

#### 4.3.2. Method Comparison

We can generally observe significant improvements in favor of the P2R and P2P methods as opposed to RandomizedBins and CloudPose for the orientation regression. The errors are reduced by around 96% for all metrics on all object types individually and combined compared to RandomizedBins. An example of this is the average MAE and RMSE for ALL, where the MAE is 10.55° and the RMSE is 13.81° for RandomizedBins. For P2P, the MAE is 0.53°, and the RMSE is 0.76°. From the error levels of CloudPose to our P2R and P2P methods, we can observe an error reduction of around 50%. One example for this is the roll for BMAE on ALL, which drops from 2.41° to 1.21°. A similar reduction can be seen between the average MAE error for ALL, which is 0.96° for CloudPose and 0.52° for P2R.

Generally, P2R and P2P result in comparable errors. On NORTH, MAV, EVS, and ALL, we observe close errors for almost all axes individually and on average. For example, the averages for MAV on the MAE are 0.47° for P2P compared to 0.46° for P2R. Similarly, the BMAE is 0.74° for P2P and 0.71° for P2R. One exception is BMAE for NORTH, were P2P performs better with an average of 1.68° against 2.47° for P2R. Despite the errors being close for HOLO, P2R performs better on HOLO in general on most measured errors. In conclusion, P2P and P2R achieve similar results, while P2P performs more consistent as observable in our NORTH example.

### 4.4. Position Results

Table 4 shows the positional $L_{2}$ error of the P2R and P2P methods on all individual axes and in total.

The extension of RandomizedBins with translation estimation does generally result in high errors. The errors are in the decimeter range on the x and y axes and the overall $L_{2}$ error. Only on the z-axis, the results stay in the centimeter range. The pattern of NORTH performing worst among all glasses continues for the position. HOLO performs best again with an $L_{2}$ error of 852.51 mm.

CloudPose achieves position errors between 4.28 mm and 7.86 mm on the z-axis. Equivalently to the z-axis, the errors on the y-axis and the $L_{2}$ error have low variation between the models. The x-axis shows errors betwen 11.20 mm for NORTH and 4.91 mm for MAV, showing higher errors for smaller glasses models.

As P2R does not regress the position, we show the point cloud estimator’s position results. For P2R, the position error on the x- and y-axis are similar, ranging from 3.59 mm to 4.14 mm on the x-axis and from 2.59 mm to 3.29 mm on the y-axis. The error is the lowest on the z-axis, ranging from 1.38 mm for HOLO to 1.54 mm for MAV. The model ALL presents a similar pattern for the orientation, where the error is in the mid-range compared to the other objects individually.

For P2P, the position estimation is similar on all four objects. Regarding the individual axes, we observe errors in similar ranges. The values for the individual axes are between 2.17 mm and 3.50 mm. The overall $L_{2}$ distance starts from 5.07 mm for EVS and ranges to 6.15 mm for NORTH. The outcome for ALL is in the mid-range compared to all individual glasses separately.

P2P mostly delivers the best position estimation. For the x and y axes and the $L_{2}$ distance, P2P brings the most notable performance improvement. On the contrary, P2R attains the best outcomes consistently on the z-axis, where the errors are between 1.66 mm and 2.24 mm.

### 4.5. Discussion

For the orientation, we observe major improvement overall from the image- and RvC-based RandomizedBins method to the point cloud-based CloudPose and our P2R and P2P methods. We can also see better estimation results with point cloud-based methods for extreme poses, which the BMAE metric shows consistently. Compared to the CloudPose method, our approaches bring improvements in orientation and position estimation. Another observation for the individual objects is the lower error for larger objects. HOLO results in the overall lowest errors for all methods, whereas NORTH results in the highest error. Between the P2R and the P2P estimation, small differences are observable. Still, the P2P method estimates a slightly better orientation, also for extreme poses. This is visible on the BMAE metric. For position regression, P2P performs best among all methods. The values on the y-axis and the $L_{2}$ error on all axes at once are lower most of the time. One exception is the z-axis, where P2R performs better.

The RvC method RandomizedBins trained on the HMDPose dataset shows acceptable results for orientation, despite resulting in considerably higher errors in contrast to the point cloud-based methods. One explanation is that it is the only image-based method and does not work with point clouds as an intermediate representation. The intermediate point cloud representation ensures an increased pose estimation accuracy for the other approaches. Another reason is the strong frontal orientation of the driver while driving, which leads to a vast amount of images and associated pose labels in similar ranges. This may make it harder to train for RvC approaches, which rely on predefined classes of pose ranges. Our extension of the method with the goal of position estimation shows poor performance. This may stem from the position not being on a continuous scale like the orientation, making the classes for RvC hard to design in an overall fitting way. This is easier for orientation, as the approach trains on Euler angles, which consists of a finite space. Additionally, the inputs of RandomizedBins in the original work are RGB images instead of IR images. The original work also focuses on head pose estimation in contrast to object pose estimation, which we conducted in this work.

Furthermore, P2R shows mostly close results to P2P, making it a good option for efficient deployment due to less computation requirement. P2P is preferable in case of better resource availability to improve estimation accuracy.

## 5. Conclusions

This paper introduced two AR glasses pose estimation methods based on point clouds generated with single infrared images. We developed and utilized a point cloud estimator custom built for multi-view infrared images to generate point clouds for estimation.

Our networks PointToPose and PointsToRotation, trained on a generated point cloud dataset, outperform a state-of-the-art algorithm on the HMDPose dataset. Generating point clouds first from images has proven useful, as we observe a significant boost in pose estimation prediction. Compared to the image-based approach RandomizedBins, we reduce the pose estimation error by around 96%. In comparison to the point cloud-based pose estimation method CloudPose, we achieve an error reduction of around 50%. The generation of point clouds and the subsequent similarity in pose estimation errors for individual and the combination of glasses point towards generalizability. In case of an in car deployment of new glasses, this can be beneficial for other, unseen types of AR glasses used while driving. We show that low resolution point clouds generated from low-cost IR hardware result in high pose estimation accuracy. In future work, we plan to investigate high resolution point clouds from depth sensors to directly compare the effect of the input point cloud quality on the pose estimation accuracy and the generalization property to different AR glasses or subjects.

## Figures and Tables

**Table 1:** Parameters for backbone layers. For each layer, we show the number of points, the ball radius, the number of nodes per layers of the PointNet and normalization information.

Layer	Num. Points	Ball Radius	PointNet	Normalization
SA1	1024	0.02	[3, 64, 64, 128]	True
SA2	512	0.04	[128, 128, 128, 256]	True
SA3	256	0.08	[256, 128, 128, 256]	True
SA4	128	1.0	[256, 128, 128, 256]	True
FP1	-	-	[512, 256, 256]	-
FP2	-	-	[512, 256, 256]	-

**Table 2:** Overview of the input and output for the backbone layers.

Layer	Input XYZ	Input Features	Output XYZ	Output Features
SA1	(B, 2000, 3)	None	(B, 1024, 3)	(B, 128, 1024)
SA2	SA1 out XYZ	SA1 out Features	(B, 512, 3)	(B, 256, 512)
SA3	SA2 out XYZ	SA2 out Features	(B, 256, 3)	(B, 256, 256)
SA4	SA3 out XYZ	SA3 out Features	(B, 128, 3)	(B, 256, 128)
FP1	SA3 out XYZ SA4 out XYZ	SA3 out Features SA4 out Features	(B, 256, 3)	(B, 256, 256)
FP2	SA2 out XYZ SA3 out XYZ	SA2 out Features FP1 out Features	(B, 512, 3)	(B, 256, 512)

**Table 3:** Orientation results of the P2R and P2P approaches compared to RandomizedBins and CloudPose on the given error metrics in degrees. The Everysight Raptor is referenced as EVS, Hololens 1 as HOLO, North Focal Generation 1 as NORTH and the Mini Augmented Vision glasses as MAV. ALL stands for all glasses combined. The roll, pitch, yaw and the average of all three axes are given on the defined metrics. The lowest values among all methods per metric on each individual axis is highlighted.

Glasses Type	Metric	RandomizedBins [6]				CloudPose [7]				P2R (Ours)				P2P (Ours)			
		Roll	Pitch	Yaw	Avg	Roll	Pitch	Yaw	Avg	Roll	Pitch	Yaw	Avg	Roll	Pitch	Yaw	Avg
EVS	MAE	4.34	3.96	21.40	9.90	0.93	0.81	1.18	0.97	0.41	**0.37**	**0.53**	**0.43**	0.46	0.38	**0.53**	0.46
	RMSE	5.28	4.95	26.42	12.22	1.46	1.19	1.52	1.39	**0.60**	**0.53**	0.87	0.66	0.64	0.53	**0.79**	**0.65**
	BMAE	16.79	10.19	35.78	20.92	6.52	0.91	1.69	3.04	**0.74**	**0.48**	2.10	1.11	1.28	0.64	**0.79**	**0.90**
MAV	MAE	5.30	8.60	20.24	11.38	0.90	0.65	1.34	0.96	**0.48**	**0.38**	**0.52**	**0.46**	**0.48**	0.39	0.54	0.47
	RMSE	6.07	9.65	25.88	13.87	1.21	0.87	1.99	1.36	0.71	**0.53**	**0.81**	0.69	**0.66**	0.54	**0.81**	**0.67**
	BMAE	14.09	12.11	33.56	19.92	2.28	0.82	3.04	2.05	**0.83**	**0.51**	0.79	**0.71**	0.91	0.63	**0.69**	**0.74**
HOLO	MAE	10.41	5.52	15.46	10.46	0.88	0.77	1.03	0.90	**0.43**	**0.34**	**0.49**	**0.42**	0.47	0.37	0.53	0.46
	RMSE	11.02	7.22	22.53	13.59	1.20	1.10	1.44	1.25	**0.61**	**0.50**	**0.75**	**0.62**	0.65	0.52	0.77	0.65
	BMAE	14.81	13.51	35.65	21.32	2.12	0.83	1.48	1.48	**0.61**	**0.40**	1.12	**0.71**	0.90	0.44	**0.82**	0.72
NORTH	MAE	4.40	7.93	15.97	9.44	1.05	0.78	1.52	1.12	**0.50**	**0.55**	**0.59**	**0.55**	0.55	0.57	0.63	0.58
	RMSE	5.30	9.89	23.17	12.79	1.41	1.11	1.83	1.45	0.78	**0.86**	0.95	0.87	**0.76**	0.87	**0.91**	**0.84**
	BMAE	11.87	20.19	39.80	23.95	2.11	**1.72**	2.09	1.98	2.80	2.58	2.04	2.47	**1.29**	2.66	**1.09**	**1.68**
ALL	MAE	3.43	10.36	17.87	10.55	1.20	0.90	0.78	0.96	**0.51**	**0.46**	**0.58**	**0.52**	0.53	0.47	0.61	0.53
	RMSE	4.46	12.41	24.55	13.81	1.48	1.13	1.12	1.24	**0.73**	0.69	**0.88**	0.77	**0.73**	**0.68**	**0.88**	**0.76**
	BMAE	17.17	18.81	48.49	28.15	2.41	0.99	1.72	1.71	**1.21**	**0.56**	1.50	**1.09**	1.81	**0.56**	**1.26**	1.21

**Table 4:** Results for the positional, Euclidean error on both approaches compared to RandomizedBins and CloudPose in millimeters. The Everysight Raptor is referenced as EVS, Hololens 1 as HOLO, North Focal Generation 1 as NORTH and the Mini Augmented Vision glasses as MAV. ALL stands for all glasses combined.ree axes are given on the defined metrics. The lowest values among all methods per metric on each individual axis is highlighted.

Glasses Type	RandomizedBins [6]				CloudPose [7]				P2R * (Ours)				P2P (Ours)			
	x	y	z	*L*_2_	x	y	z	*L*_2_	x	y	z	*L*_2_	x	y	z	*L*_2_
EVS	794.96	483.75	27.48	932.43	10.90	8.06	5.65	15.86	3.68	3.11	**1.40**	5.66	**2.95**	**2.23**	2.64	**5.26**
MAV	754.36	510.57	48.21	914.04	4.91	7.29	4.28	11.06	3.80	2.68	**1.54**	5.49	**2.86**	**2.17**	2.55	**5.07**
HOLO	740.35	415.94	51.87	852.51	7.87	7.43	7.86	14.81	3.59	3.29	**1.38**	5.71	**3.03**	**2.32**	2.65	**5.35**
NORTH	837.18	595.99	40.53	1029.78	11.20	6.09	5.92	15.85	4.14	2.59	**1.40**	**5.66**	**3.50**	**2.51**	3.15	6.15
ALL	748.66	473.33	41.27	887.97	7.83	7.87	7.76	15.28	3.85	2.92	**1.40**	**5.68**	**3.24**	**2.38**	2.96	5.75

* As P2R doesn’t regress the position, we show the position results taken from the point cloud estimator.

## Data Availability

Data available in a publicly accessible repository that does not issue DOIs Publicly available datasets were analyzed in this study. This data can be found here: https://ags.cs.uni-kl.de/datasets/hmdpose/.
